# Agricultural Landscapes and Forest Fragments Shape Population Dynamics, Cuticular Lipids and the Conservation of the Masked Bees, *Hylaeus communis*

**DOI:** 10.1002/ece3.74436

**Published:** 2026-10-05

**Authors:** Sara Olberz, Steven Jansen, Manfred Ayasse, Samuel Boff

**Affiliations:** ^1^ Institute of Evolutionary Ecology and Conservation Genomics Ulm University Ulm Germany; ^2^ School of Life Sciences Technical University of Munich Freising Germany; ^3^ Institute of Botany Ulm University Ulm Germany; ^4^ Laboratory for Bees Studies Federal University of Maranhão São Luís Brazil

**Keywords:** conventional farming matrix, cuticular hydrocarbons, *Hylaeus*, organic farming, sex ratio

## Abstract

Organic farming has been shown to offer advantages over conventional farming in protecting insect biodiversity, but our overall understanding of its relationship with wild bee population parameters, especially cuticular lipids, remains limited. In the current study, we installed bee hotels at different farming sites in southern Germany and hypothesized that the local farming system (organic vs. conventional), the extent of organic farming, and the extent of forest (percentage of each component in the analyzed landscape) influence the abundance of nests and individuals of small‐bodied solitary bees of the genus *Hylaeus*. Moreover, at the species level, we investigated the influence of the same agricultural landscape elements and forest cover on the number of nests, bee abundance, sex ratio, and body size of *H. communis* , the most common *Hylaeus* species at our study sites. We further investigated how this landscape variables influence the composition of cuticular lipids in females and males of this species. Our results were more conclusive at the species level. We found that organic farming positively influenced the abundance of *H. communis* , whereas abundance decreased with increasing forest cover. In contrast, the sex ratio was higher at conventional farming sites and increased with forest cover. Although females were larger than males, no effect of landscape variables on body size was detected. The chemical composition of cuticular lipids of *H. communis* was significantly influenced by the farming system. These findings raise concerns about potential mechanisms through which farming practices may influence the reproduction of cavity‐nesting wild bees in agricultural environments. In conclusion, our findings show a positive effect of organic farming on the abundance of *H. communis* , while also highlighting potential effects of farming practices on physiological traits and population‐level parameters of this small wild bee species.

## Graphical abstract

Small size solitary bees are often neglected. We successfully sample tiny masked bees with trap nests. We found population living in conventional farm lands are under risk compared to organic farm lands.

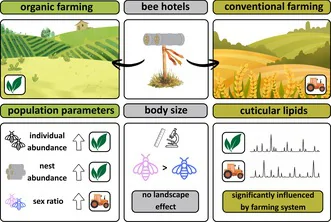

## 1 Introduction

The natural contribution of wild bees to pollination services is crucial for wild and crop plants (Buchmann and Ascher 2005; Fründ et al. 2013; Garibaldi et al. 2013; Potts et al. 2010). Thirty‐five percent of global crop production (Klein et al. 2007) and the pollination of 84% of European crop species (Williams 1994) are provided mostly by bees (Allen‐Wardell et al. 1998; Giannini et al. 2015). Despite the importance of bees, considerable declines in bee abundance and species richness (Ghisbain et al. 2024; Patiny et al. 2009) have been observed globally (Kearns et al. 1998; Zattara and Aizen 2021), especially in agricultural areas (Raven and Wagner 2021; Turley et al. 2022). These declines are often overlooked when considering solitary bees and farming management, especially the recently found, potentially greater effects on small‐bodied species (Maas et al. 2021; Schultz et al. 2026).

Previous studies focusing on the influence of farming management on bees comparing the effects of conventional and organic farming, have provided evidence that organic farming enhances habitat quality (Kennedy et al. 2013; Power and Stout 2011). It is also known to favor to some extent the presence of arthropods like spiders (Feber et al. 1998), wasps (Somavilla et al. 2016) and honeybees (Wintermantel et al. 2019). In solitary bees, the influence of farming management and associated landscapes are mostly investigated on *Osmia* (Boff et al. 2024; Gruber et al. 2011; Holzschuh et al. 2008; Martínez‐Núñez et al. 2020; Williams and Kremen 2007). Although small wild bee species are of global interest due to their importance as pollinators (Kuppler et al. 2023), our overall knowledge on the influence of farming management on small wild bees compared to other bees is still poor (but see: Benjamin et al. 2014; Grab et al. 2019). Thus, adding information about small wild bees regarding their nesting frequency (Potts et al. 2010), abundance (Fründ et al. 2013), sex ratio (Stuligross and Williams 2021) and body size (Greenleaf et al. 2007) can make an important contribution to the conservation of small wild bees in agricultural dominated landscape. Moreover, expanding our knowledge of the composition and variability of individual cuticular lipids allows us to better understand how stressful events affect the physiology of small solitary bees (Boff and Ayasse 2024).

Cuticular lipids evolved as crucial primary component for avoiding body dehydration and chemical for communication in bees (Ayasse et al. 2001). Specific cuticular lipids or mixtures are important in the social determination of caste and age in social bees (Nunes et al. 2009), nest recognition in wild bees (Vandenabeele and Schmitt 2023) and regulation of reproduction in primitively eusocial bees (Steitz and Ayasse 2020). Furthermore, cuticular lipids drive sex communication by triggering the copulatory repertoire necessary for male and female wild bees to engage in sexual reproduction (Boff et al. 2022, 2024; Ganuza et al. 2022). Although it is known that stress affects the production of cuticular lipids (Conrad et al. 2010; Boff et al. 2022), only a few studies—on bumble bees and bigger size cavity nesting bees—have investigated the extent to which the agricultural landscape can influence cuticular hydrocarbons (Boff et al. 2024; Straub et al. 2023).

The use of bee hotels in agricultural environments and the resulting high number of captured masked bees (Colletidae: *Hylaeus*) allowed us to investigate how a small wild bee responds to local farming management (organic vs. conventional) and the associated landscape. Here, we monitored the number of bees of the genus *Hylaeus* emerging from nests established by founder females in agricultural fields and quantified the number of occupied nests and individuals as measures of bee abundance. We further assessed landscape composition surrounding the study sites to determine how the extent of organic farming (percentage of organic farming in the conventional agricultural matrix) influences population parameters of these bees. We hypothesized that farming management influences the availability and diversity of floral resources in meadows surrounding bee hotels. Moreover, we tested how farming management (local scale), forest cover and agricultural landscapes affect the number of *Hylaeus* nests and emerging bees. Meanwhile forest cover (extent of forest) was expected to influence these responses through changes in resource availability in the surrounding landscape. At the population level, we used *H. communis* , the most common *Hylaeus* species in Europe (Dathe 2000) and the only one consistently present across sexes and study sites. The effects of farming management (organic vs. conventional) and landscape composition (extent of organic farming and forest) on abundance, sex ratio, body size and cuticular lipid profiles of *H. communis* were investigated. Specifically, we hypothesized, based on previous studies of mason bees, that (I) the abundance of *H. communis* would be higher under organic farming. (II) Farming system and forest cover would influence sex ratio. (III) *Hylaeus* is likely to depend strongly on resources in its immediate surroundings as a small wild bee, hence the body size of *H. communis* would vary with sex, farming system (organic vs. conventional) and the extent of organic farming. (IV) The composition of cuticular lipids would differ between farming systems and sexes, including sex‐specific responses to farming management.

## 2 Material and Methods

### 2.1 Agricultural Sites and the Genus *Hylaeus*

The study took place on 17 different farms in Baden‐Württemberg, southern Germany, of which nine were organic and eight were conventionally managed (Figure 1). Each farming site was equipped with a total of eight bee hotels (except site number two, which had three stations and six nests), two were always set up together per station between cereal fields and meadows.

**FIGURE 1:**
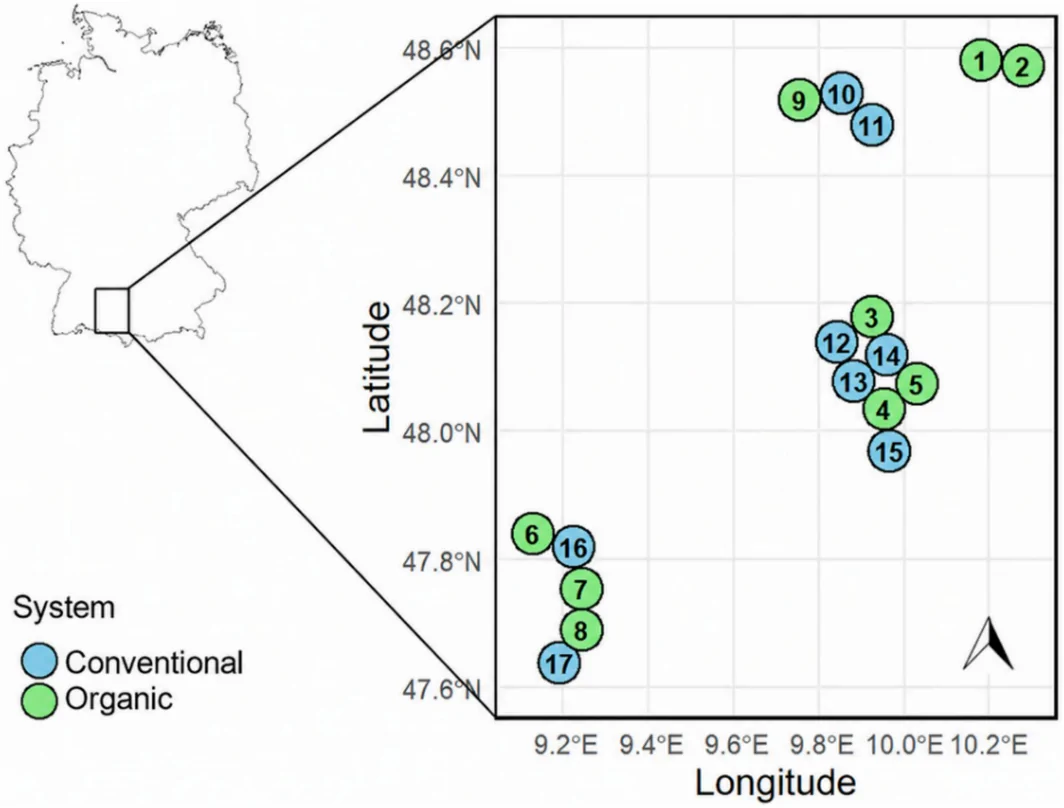
Map of Germany (left side) with a marked section of the study region in Baden‐Württemberg where the study was conducted. Each site included four stations with eight bee hotels each site (except number two, which had six). Nine labels in green (1–9) represent organic and eight blue labels represent conventional farms (10–17).

The trap nests consisted of PVC pipes (100 mm i.d.) filled with *Phragmites* reeds with randomized sizes (200 mm × 4–12 mm i.d.), offering empty cavities for *Hylaeus* females (founders) to build new nests (Staab et al. 2018; Westerfelt et al. 2018). The bee hotels were protected from sun and rain by a wooden board on top serving as a roof. Their installation at the faming sites took place in April 2021, allowing the bees to nest there for approximately six months. Then in September of the same year, the bee hotels were removed from the field and occupied nests were individually enclosed inside a labeled piece of tubing (220 mm × 1.5 mm i.d.) with cotton balls at both ends to ensure air supply for developing bees. All nests were moved to the greenhouse on the roof of the university building in Ulm (Germany) and remained there under natural temperature conditions. During this time, nests were carefully opened, and the number of brood cells was counted before the bees emerged. At the beginning of winter (November of 2021), the nests were placed in a climate chamber of the university's institute at a controlled temperature of 4°C to overwinter there. At the end of February 2022, the nests were returned to the university's roof until the adult bees emerged. After emergence, *Hylaeus* bees were identified to the species level. Due to its relatively large sample size, individuals of *H. communis* were transferred to 1.5 mL Eppendorf Safe‐Lock tubes and stored at ‐ 80°C until extraction of the cuticular lipids with pentane, as described below. After extraction of cuticular lipids, *H. communis* bees were measured for body size.

### 2.2 The Genus *Hylaeus*

The genus *Hylaeus* (Fabricius, 1793), comprising small wild bees commonly known as masked bees. In Germany, they consist of 45 solitary species (Westrich 1989). These bees are characterized by their wasp‐like appearance with hairless black bodies and a distinctive white or yellow facial pattern. In nature, they nest in empty cavities like plant stems or hollow tree trunks and build nests using silky, membranous material to close off each brood cell (Batra 1972; Fye 1965; Westerfelt et al. 2018). In the current study, we performed genus‐level analyses of *Hylaeus* by first pooling all species and then conducting population level species‐specific analyses of *H. communis* , which had the highest number of nests. *H. communis* , the most widespread species in Europe (Dathe 2000) is a pronounced generalist, pollinating a wide range of plants (Casanelles‐Abella et al. 2022; Müller 2023; Ruszowski and Gosek 1999). Despite its importance for plant pollination, *H. communis* has rarely been studied especially at the population‐level and regarding chemical ecological responses to local farming management and the associated landscape.

### 2.3 Landscape and Floral Analysis

Landscape composition was quantified within circular areas with a 500 m radius centered on each bee hotel. This radius was selected to encompass the expected foraging range and maximum flight distance of small cavity‐nesting bees (Gathmann and Tscharntke 2002). The landscape surrounding the bee hotels consisted predominantly of agricultural land under organic or conventional management, interspersed with forest. Land use was characterized by using the same procedure for both organic and conventional sites. First, each distinguishable land‐use unit within the 500 m radius was delineated separately using the polygon tool in Google Earth Pro based on 2021 Landsat/Copernicus imagery. Second, the study sites were visited to verify the land‐use classification of the mapped areas. Finally, local farmers were consulted to confirm whether individual agricultural units were managed organically or conventionally. Thus, the extent of the agricultural management within the surrounding landscape was determined independently of the classification of the agricultural site.

Within each 500 m foraging area, we initially quantified the extent of organic farming, conventional farming, and forest cover. The total agricultural area included both organically and conventionally managed agricultural land. The extent of organic farming was calculated as the summed area (m^2^) of organically managed cropland, meadows, and grasslands divided by the total agricultural area under organic and conventional management within the 500 m radius. The extent of conventional farming was calculated analogously. Forest cover was calculated as the proportion of the entire circular area occupied by forest.

Landscape composition surrounding each agricultural site was characterized by the proportional cover of organically as well as conventionally managed agricultural land, and forest. Conventional sites occurred in landscapes with mean proportions of 0.721 conventional farming, 0.066 organic farming, and 0.211 forest cover, whereas organic sites occurred in landscapes with mean proportions of 0.235 conventional farming, 0.676 organic farming, and 0.284 forest cover. Thus, the main contrast between farming systems was associated with the relative proportions of organic and conventional agricultural land in the surrounding landscape, while forest cover was comparatively similar between the two groups.

Prior to statistical modeling, we evaluated the relationship between the extent of organic and conventional farming. These variables were strongly negatively correlated (Pearson's *r* = −0.924, *p* < 0.001, *n* = 17). To avoid the simultaneous inclusion of highly correlated predictors, we retained the extent of organic farming as the landscape‐scale agricultural management variable because it directly represented the component relevant to our primary hypothesis. Forest cover was retained as a separate explanatory variable representing the availability of semi‐natural habitat within the potential foraging area.

Flower species and abundance were monitored only once during the summer of 2021. The counting occurred within a radius of 100 m around the bee hotels. Two plots of 25 m^2^ each were analyzed in each direction (N, E, S, W), covering a total area of 200 m^2^. To randomize the distribution of plots, an ordinary app was used to randomly selected a number between 0–50 and 51–100 m. This was set as the distance from the center point, meaning the location where the bee hotels were installed (Figure 2). The flower abundance was calculated based on the sum of all flowers present in the plots. In contrast, the flower diversity was calculated based on the average number of flowering species (plant species) in these plots to consider species composition. The foraging distance of *Hylaeus*, as seen in other small bees, is usually up to about 200 m (Gathmann and Tscharntke 2002), so a fair proportion of the foraging area was covered by the floral analysis.

**FIGURE 2:**
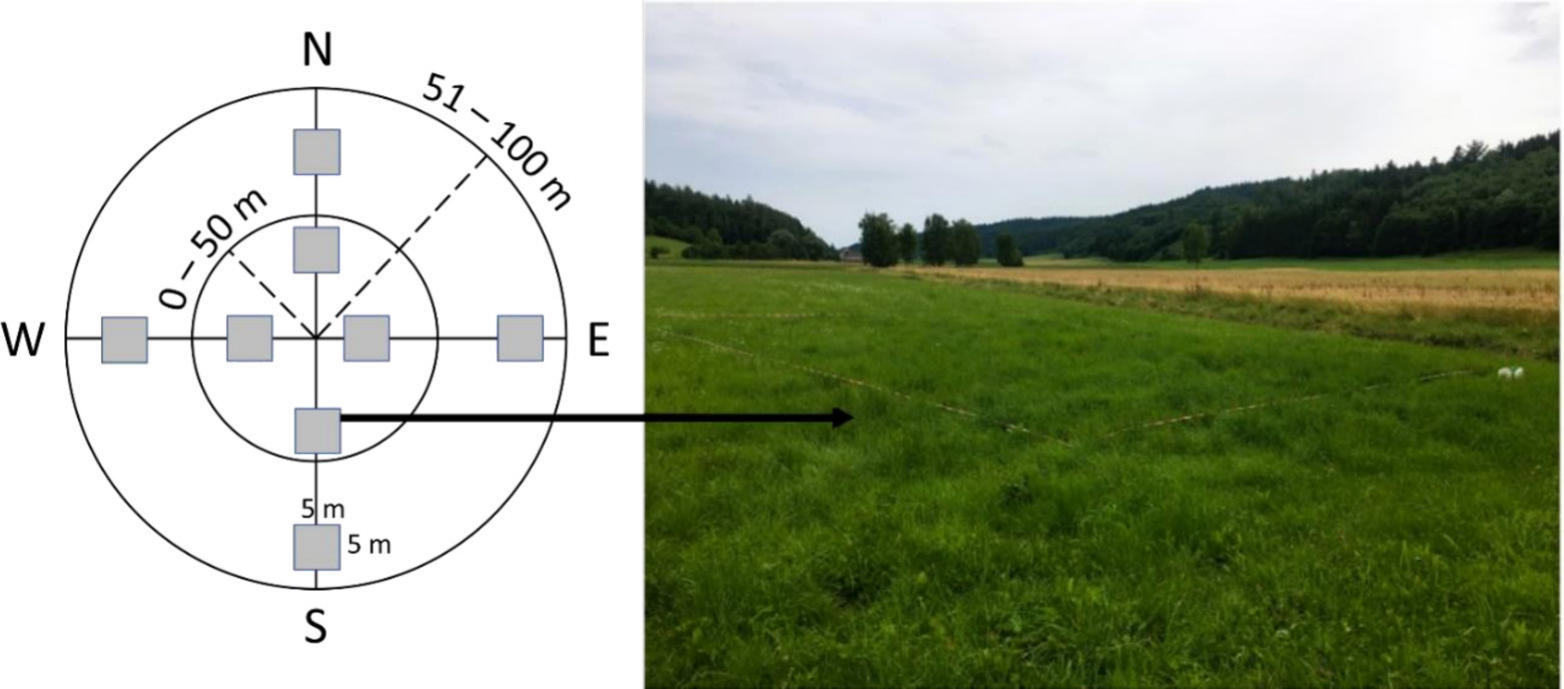
Scheme of the floral analysis in a randomly distributed plot of 25 m^2^ (5 × 5 m) within a radius of 100 m around the center point, which represents the location of eight bee hotels per farming site. One plot was placed in each direction (N, E, S, W) at 0–50 and 51–100 m.

### 2.4 Determination of Sex and Species

Initial analyses were performed by pooling all data on nests and bees of the genus *Hylaeus*, regardless of species. Nonetheless, all *Hylaeus* species collected were identified to the species level using the identification key “Fauna Helvetica 4: Apidae 2” by. To assess differences in the number of nests and emerging bees, we pooled the data from all *Hylaeus* species. Further analyses were conducted on *H. communis* to assess body size, sex ratio and the composition of cuticular lipids.

### 2.5 Cuticular Lipids of *H. communis*

To extract the cuticular lipids, the individual bees were put into glass vials with a volume of 1.5 mL and washed in 300 μL of n‐pentane (SUPRASOLV, purity ≥ 99.9%). The cuticular lipids were dissolved by shaking the vials using an OHAUS AnalogVortexMixer for three minutes. Then, the bees were removed from the vials and the extracts were stored at −80°C in a freezer. Before the chemical analysis with the Agilent Technologies 7890B gas chromatograph (GC), the sample volume was concentrated with a gentle flow of nitrogen to a final volume of 60 μL. Additionally, 10 μL of a 1 mg/mL stock solution of hexadecane was added as an internal standard to determine the absolute amounts of individual substances. A GC run started at an oven temperature of 50°C with a splitless injection of 1 μ*L* of sample carried out by an Agilent Technologies 7693 Autosampler. The splitter valve opened after 1 min and the oven temperature was increased by 10°C/min until it reached 310°C temperature. The highest temperature was maintained for 18 min and after a total of 45 min the GC run ended. Hydrogen served as the carrier gas with a flow rate of 2 mL/min. A J&W Scientific Folsom GC DB‐5MS Capillary Column (30 m × 0.25 mm i.d. × 0.25 μm film thickness) and a flame ionization detector (FID) were installed in the device. The gas chromatograms were evaluated with the Agilent ChemStation Software (Agilent Technologies).

The compounds were identified using a mass spectrometer. For this purpose, we used the coupled system of an Agilent Technologies 7890B GC and a 5977 MSD, including a Gerstel Thermal Desorption Unit (TDU). The running conditions were the same as those mentioned above for the GC system. These compounds were additionally checked against reference mixtures of synthetic compounds including alkanes and alkenes in the GC and assigned to the corresponding peaks based on their retention times. To identify the double bond positions of the alkenes, derivatization with dimethyl sulphide (DMDS) was carried out (Schmitt, personal communication).

### 2.6 The Body Size of *H. communis*

Every single bee was placed in a wax mold on a glass slide (Figure S1) under a Zeiss Stemi 508 stereomicroscope connected to an Axiocam 208 color camera. The Zeiss Efficient Navigation (ZEN) 3.2 (blue edition) software was used to measure the intertegular distance (ITD), which extends between the wing bases (Cariveau et al. 2016). This standard measure of body size is positively correlated with the dry weight of bees (Cane 1987) and other body measurements (Nooten and Rehan 2020).

### 2.7 Statistical Analysis

Data analyses were conducted using R version 4.4.2 and RStudio 2024 using the lme4 (Bates et al. 2015), glmmTMB (Brooks et al. 2017), DHARMa (Hartig 2016), vegan (Oksanen et al. 2001), MuMIn (Barton 2013), and ggplot2 (Wickham and Sievert 2009) packages. Differences in floral resources between farming systems were assessed separately for flower species richness and flower abundance. Average flower species richness was analyzed using a Gaussian generalized linear model (GLM), with farming system (organic vs. conventional) as the explanatory variable. Total flower abundance, which included zero values and showed a strongly right‐skewed distribution, was analyzed using a GLM with a Tweedie distribution and a log‐link function.

To investigate population traits of *Hylaeus*, three explanatory variables were considered: (1) farming system, to compare organic and conventional farming; (2) extent of organic farming, defined as the proportion of organically managed area within the agricultural matrix (Table S1); and (3) extent of forest, defined as the proportion of the total area covered by forest within a 500 m radius around the bee hotels (Table S1). These explanatory variables were initially considered in the global models, whereas floral‐resource variables were not included because they were measured only once and therefore might not adequately represent resource availability throughout the nesting period. Model selection was performed using the Akaike Information Criterion corrected for small sample size (AICc), with predictors removed when their exclusion reduced AICc by at least two units. Model assumptions were evaluated using simulated residual diagnostics implemented in the DHARMa package (Hartig 2016).

At the genus level, information from all *Hylaeus* species was pooled. The number of occupied nests and the number of adult *Hylaeus* bees emerging from the nests were used as response variables. Both responses were analyzed using zero‐inflated negative‐binomial generalized linear mixed models (GLMMs; nbinom2, log link), including farming system, forest extent, and their interaction as explanatory variables, with site included as a random factor.

At the species level, we investigated abundance, sex ratio, body size, and the composition of cuticular lipids of *H. communis* . The abundance of *H. communis* was analyzed using a zero‐inflated negative‐binomial GLMM (nbinom2, log link), with farming system, forest extent, and extent of organic farming as explanatory variables and station nested within site as a random factor. Sex ratio was analyzed using a Gaussian GLM with farming system and forest extent as explanatory variables, using data pooled by site. Body size was analyzed using a linear mixed‐effects model (LMM), with sex, farming system, and extent of organic farming as explanatory variables and station nested within site as a random factor.

Differences in the composition of cuticular lipids of *H. communis* were investigated using non‐metric multidimensional scaling (NMDS) based on Bray–Curtis dissimilarities. Prior to analysis, the relative abundance of each cuticular lipid was calculated for each individual by dividing the mass concentration of each compound by the total mass concentration of all compounds in that sample. Compounds representing < 0.1% per sex and site were excluded from the analysis. Permutational multivariate analysis of variance (PERMANOVA) was used to test differences in cuticular‐lipid composition between sexes and farming systems. To investigate sex‐specific responses to farming system, separate NMDS and PERMANOVA analyses were conducted for females and males.

Model coefficients (*β*) and their 95% confidence intervals are reported as effect estimates for the univariate models. For PERMANOVA, *R* ^2^ is reported as the measure of effect size. Statistical significance was assessed at *α* = 0.05.

## 3 Results

### 3.1 Flowering Resources Surrounding Bee Hotels

The number of flowering plant species and total flower abundance (Table S2) were significantly higher at organic than at conventional farming sites. Flower species richness was higher at organic sites (mean ± SD: 5.42 ± 3.62) than at conventional sites (1.44 ± 1.31; GLM: *β* = 3.98, 95% CI [1.32, 6.64], *F*_1,15_ = 8.60, *p* = 0.010; Figure 3a). Similarly, total flower abundance was higher at organic sites [59,296 ± 85] than at conventional sites [2,207 ± 2] (Tweedie GLM: *β* = 3.29, 95% CI [1.67, 4.91], *χ* ^2^_1_ = 15.90, *p* < 0.001; Figure 3b).

**FIGURE 3:**
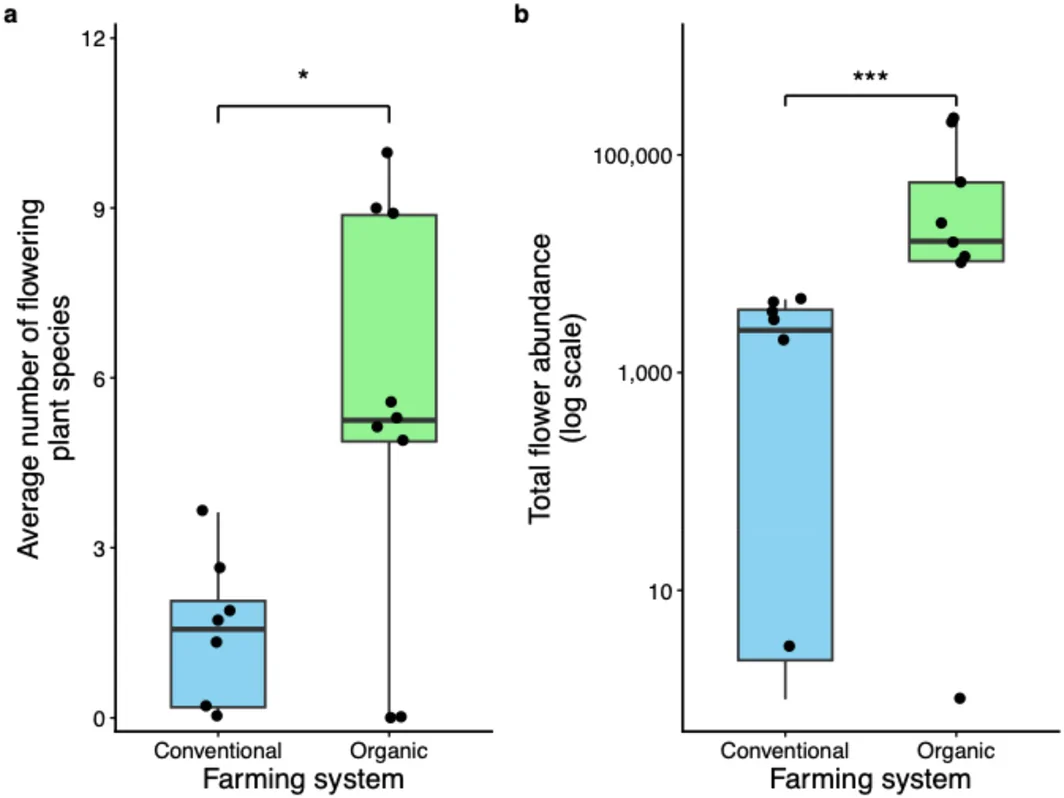
Floral resources at conventional and organic farming sites. (a) Average number of flowering plant species and (b) total flower abundance (log scale) in conventional and organic farming sites. Points represent individual sites, and the boxes show range from the first to the third quartile, representing the interquartile range (IQR), and include the median, the middle value of the data. Asterisks indicate significant differences between farming systems (*p < 0.05; ***p < 0.001).

### 3.2 Overview on *Hylaeus*

At the genus level, 57 *Hylaeus* nests were recorded at conventional farming sites, compared with 148 nests at organic farming sites (Table S3). A total of 403 *Hylaeus* bees emerged from the bee hotels, of which 105 individuals (43 females and 62 males) originated from conventional sites and 298 (123 females and 175 males) from organic sites. Despite these numerical differences, the models provided limited statistical support for effects of farming system or forest extent on the number of *Hylaeus* nests or emerged bees (Table S4; Figures S2 and S3).

### 3.3 Influence of Forest and Farming Management on *H. communis*

For *H. communis* specifically, 31 nests were recorded at conventional farming sites and 53 nests at organic farming sites (Table S3). The abundance of *H. communis* decreased significantly with increasing forest cover (GLMM: *β* = −2.80, 95% CI [−5.16, −0.43], *χ* ^2^ = 5.36, df = 1, *p* = 0.021; Figure 4a) and was significantly higher at organic farming sites (115 ± 12.9) than at conventional sites (59 ± 7.38; GLMM: *β* = 1.69, 95% CI [0.31, 3.07], *χ* ^2^ = 5.72, df = 1, *p* = 0.017; Figure 4b). The abundance of *H. communis* was not significantly associated with the extent of organic farming (GLMM: *β* = −1.20, 95% CI [−2.99, 0.59], *χ* ^2^ = 1.72, df = 1, *p* = 0.189).

**FIGURE 4:**
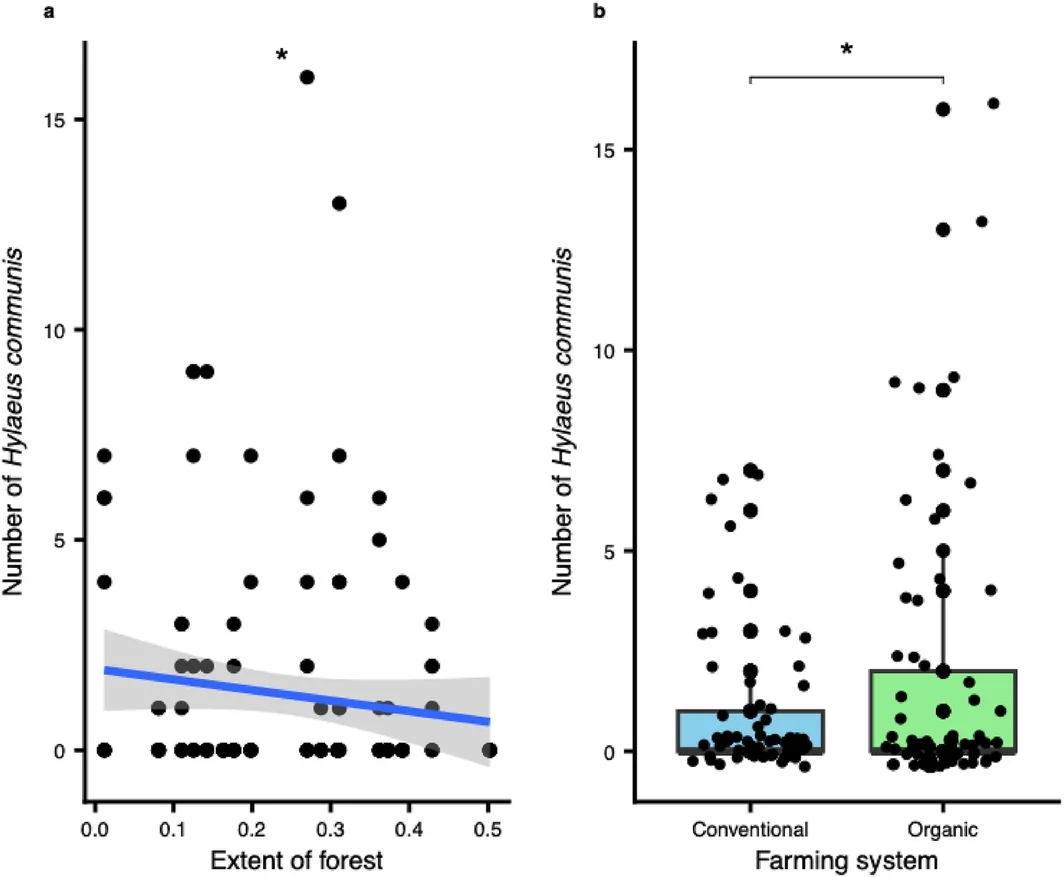
Number of *H. communis* individuals in relation to forest‐agriculture landscape elements. (a) The number of *H. communis* bees decreased with increasing forest cover and (b) the number of *H. communis* individuals was higher under the organic farming system; the box ranges from the first to the third quartile, representing the IQR, and includes the median, the middle value of the data. Asterisks indicate significant differences between farming systems (*p < 0.05).

The sex ratio of *H. communis* increased with increasing forest cover (GLM: *β* = 1.26, 95% CI [0.24, 2.28], *χ* ^2^ = 5.83, df = 1, *p* = 0.016; Figure 5a) and was significantly lower at organic farming sites (GLM: *β* = −0.35, 95% CI [−0.60, −0.09], *χ* ^2^ = 7.05, df = 1, *p* = 0.008; Figure 5b).

**FIGURE 5:**
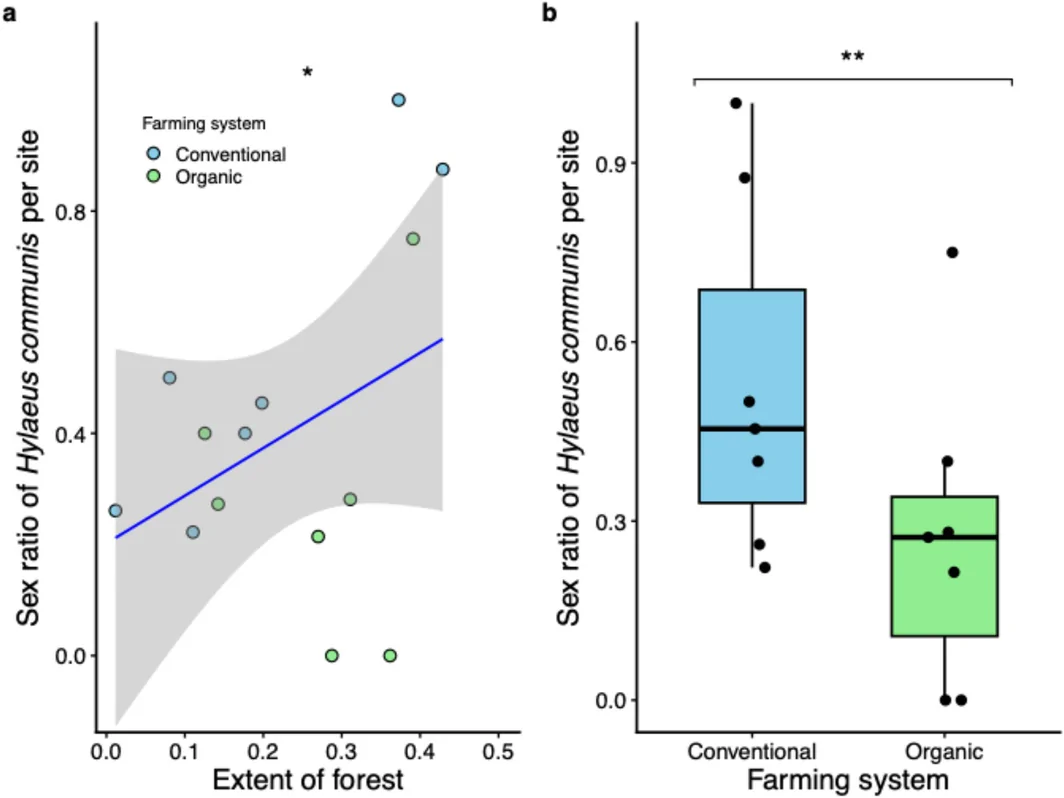
Sex ratio of *H. communis* (a) increased with the extent of forest cover and (b) was lower at organic than at conventional farming sites; the box ranges from the first to the third quartile, representing the IQR, and includes the median, the middle value of the data; the extending whiskers represent the range of variation; and the shading around the blue regression line shows the 95% confidence interval. Asterisks indicate significant differences between farming systems (*p < 0.05; **p < 0.001).

### 3.4 Effect of Farming System on Body Size of *H. communis*

A major effect of sex was detected on the body size of *H. communis* , with males being significantly smaller than females (LMM: *β* = −158.55, 95% CI [−189.36, −128.22], *χ* ^2^ = 101.79, df = 1, *p* < 0.001; Figure 6). In contrast, body size was not significantly associated with farming system (LMM: *β* = −27.60, 95% CI [−77.25, 22.17], *χ* ^2^ = 1.11, df = 1, *p* = 0.291) or the extent of organic farming (LMM: *β* = 87.65, 95% CI [−57.49, 238.96], *χ* ^2^ = 1.33, df = 1, *p* = 0.249; Table S5).

**FIGURE 6:**
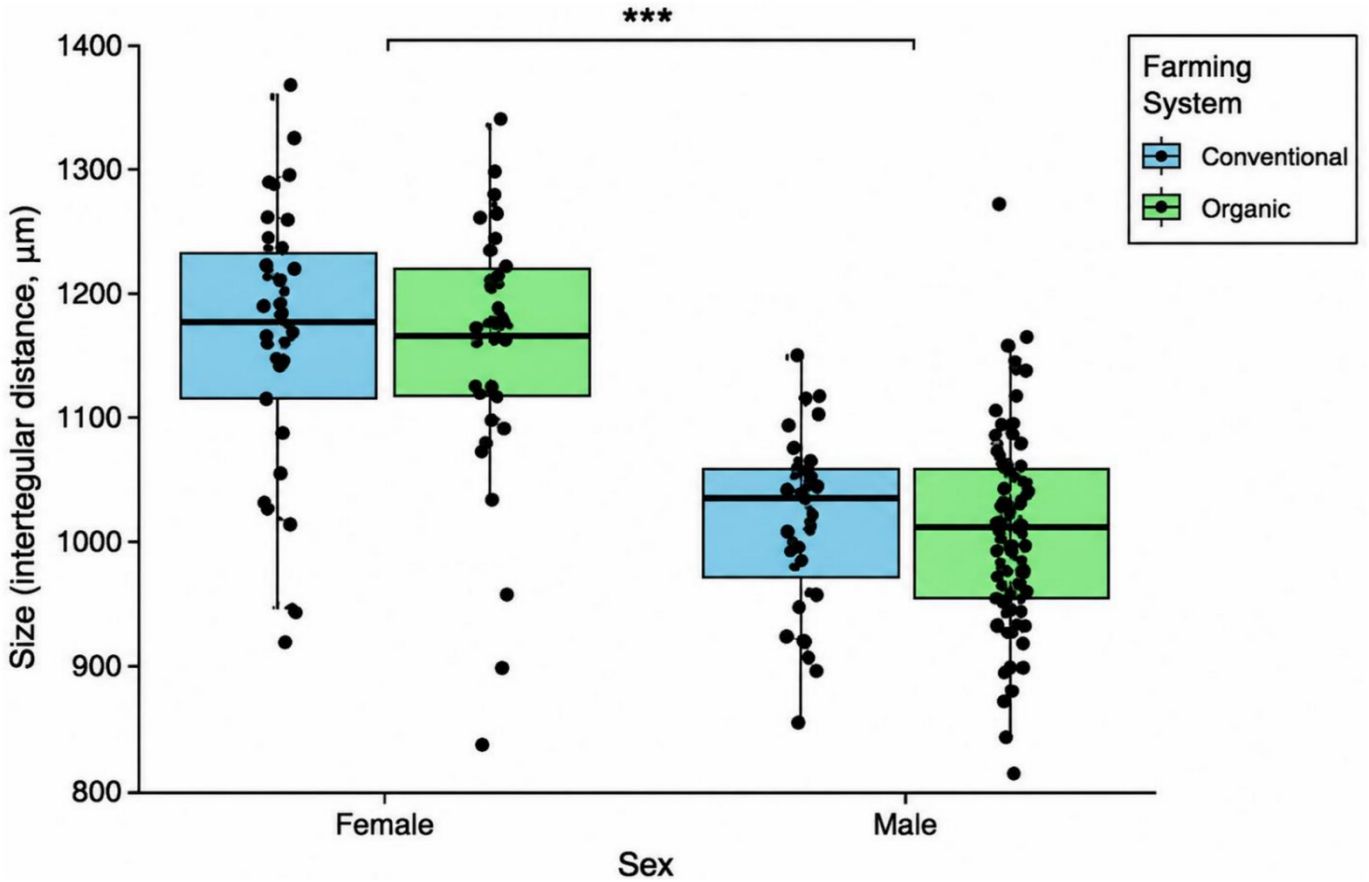
Boxplot of body size (μm) of *H. communis* , measured by the ITD, of female (*n*_conventional_ = 34, *n*_organic_ = 34), and male (*n*_conventional_ = 33, *n*_organic_ = 82) *H. communis* from organic and conventional farming sites. Only sex had a major effect on body size. The box ranges from the first to the third quartile, representing the IQR, and includes the median, the middle value of the data. The extending antennas represent the variation width. Asterisks indicate significant differences between farming systems (***p < 0.0001).

### 3.5 Effect of Farming System on the Composition of Cuticular Lipids

A total of 56 characteristic peaks (individual cuticular lipids) were detected by analyzing the extracts of *H. communis* males and females (Figure S4a,b, Table S6). Thirty‐six of these compounds were identified, including 17 alkanes, 18 alkenes and one monounsaturated fatty acid ((Z)‐9‐octadecenoic acid). Detected alkanes had C‐chain lengths between 17 and 33, whereas the C‐chains of alkenes were 21 to 31 carbon atoms long, with double bond positions at 7, 9 or 11. Unknown components ranged from presumably C13 to C33.

The pool of compounds and the composition of cuticular lipids were sex‐specific (PERMANOVA, *R* ^2^ = 0.0386, *p* = 0.005) and differed significantly between farming systems (PERMANOVA, *R* ^2^ = 0.081, *p* = 0.001; Figure S5). The centroid coordinates in the male NMDS ordination differed considerably between organic (NMDS1 = 0.0918, NMDSŚ2 = 0.1258) and conventional farming sites (NMDS2 = −0.1610, NMD2 = −0.2694). A similar pattern was observed for females, with the centroid of organic farming sites (NMDS1 = 0.1768, NMDS2 = 0.0054) clearly separated from the one of conventional farming sites (NMDS1 = −0.3633, NMDS2 = −0.0112). A significant difference in the composition of cuticular lipids between farming systems was also found for the sex‐specific analyses for both males (PERMANOVA, *R* ^2^ = 0.0855, *p* = 0.002; Figure 7a) and females (PERMANOVA, *R* ^2^ = 0.1346, *p* = 0.001; Figure 7b).

**FIGURE 7:**
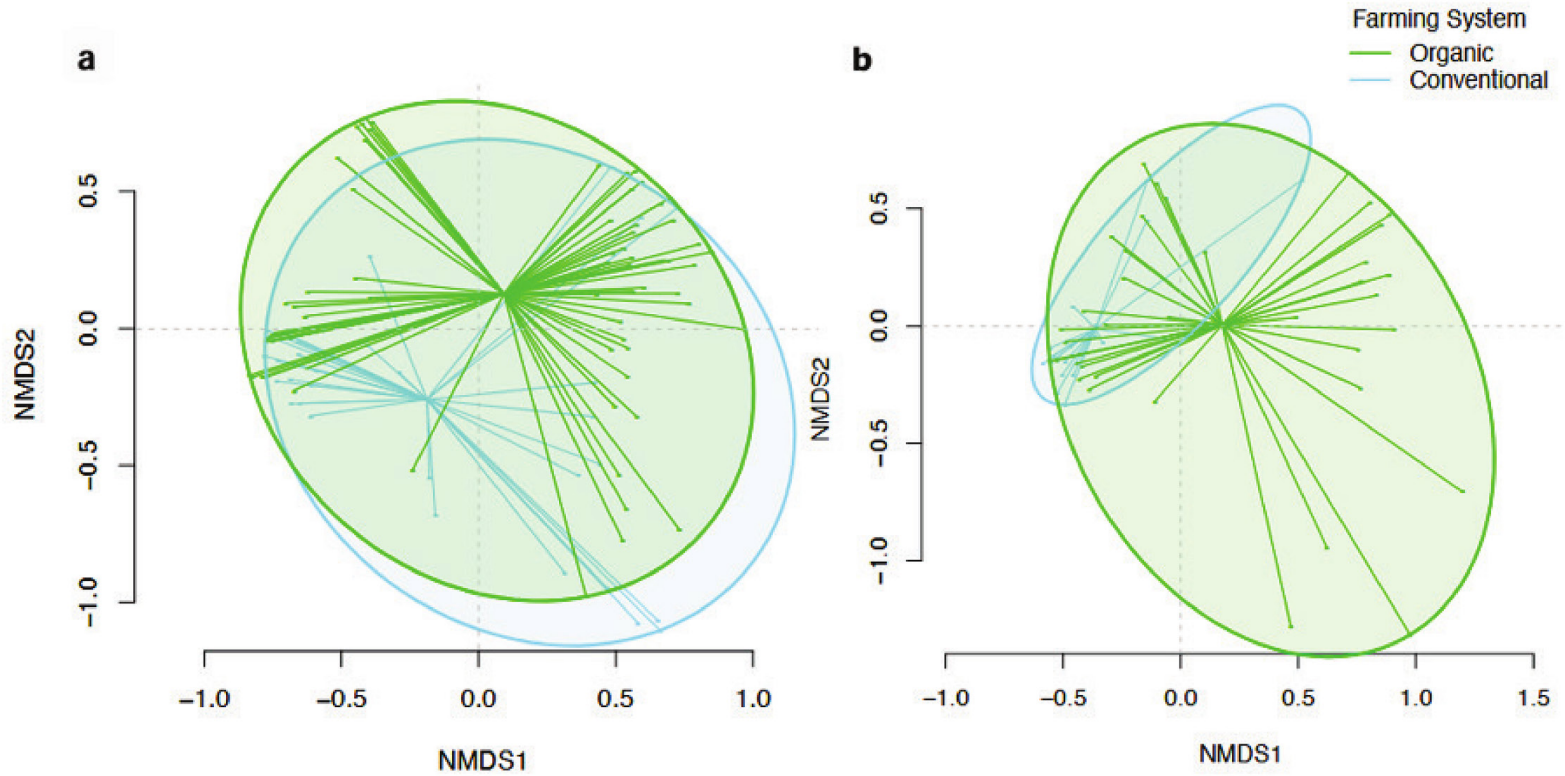
NMDS of the cuticular lipids of *H. communis* . (a) Individual bouquets of male (♂, *R* ^2^ = 0.962) and (b) female (♀, *R* ^2^ = 0.96) from organic and conventional farming sites. All coordinates are connected to the centroids of the respective groups.

## 4 Discussion

The overall analysis did not show an effect of landscape on *Hylaeus*, only a trend showed more *Hylaeus* bees on organic managed sites, implicating that different species may respond different to environmental pressures. However, we found the population of *H. communis* , the most common species in Europe, to be influenced by the agricultural landscape. We found a significant influence by the farming system (organic vs. conventional) and the extent of forest in this species. Yet, the sex ratio of *H. communis* was male biased in organic farming areas and female biased with increasing extent of forest. There were no significant correlations between body size and farming system. However, the females of *H. communis* were significantly larger than males. The composition of cuticular lipids of *H. communis* was sex‐specific and differed significantly between farming systems.

### 4.1 Effect of Farming Systems in Abundance of the Bees

The abundance of bees was meant to be higher in organic farming. Holzschuh et al. (2008) and Boff et al. (2024), for example, showed a significant decrease in bee abundance in an agricultural matrix dominated by conventional farming. Consistent with this, we were able to show a positive trend for *Hylaeus* nests and a significantly higher number of *H. communis* bees in organic farming sites. These differences are probably caused by landscape fragmentation (Schüepp et al. 2011; Zurbuchen et al. 2010), leading to habitat loss (Fahrig 2003; Smith et al. 2009), deficient food supply (Power and Stout 2011) and a lack of nesting sites (Haffmans and Ehrmantraut 2011). Furthermore, increased toxicity due to pesticide exposure (Catania et al. 2025; Albacete et al. 2024) could be another factor contributing to the decline of solitary bees in conventional farming. Although we did not test the influence of pesticides, both systems differ in terms of the type and amount of chemicals applied (BVL 2025; Kennedy et al. 2013). Based on that, it is expected that larvae are exposed to toxins present on brood diet (Stuligross and Williams 2021) and soil (Willis Chan and Raine 2021). Foragers are impaired during the collection of pollen (da Luz et al. 2018; Goulson et al. 2015; Pacífico da Silva et al. 2015), nesting materials (Danforth et al. 2019) and potentially by pesticide drift (Aude et al. 2003).

Our study focused on cavity‐nesting bees sampled using bee hotels. However, similar positive responses to organic farming have been reported using other sampling approaches and for bee communities with different nesting strategies. Studies based on flower‐visiting bees have reported greater bee abundance or diversity under organic farming (Holzschuh et al. 2008; Power and Stout 2011). Similarly, Forrest et al. (2015) found higher bee species richness and abundance on organic than conventional farms in communities in which below‐ground nesting bees were strongly represented. A broader synthesis across crop systems also showed that organic and diversified farming practices can support wild bee abundance and richness (Kennedy et al. 2013). Thus, while our findings specifically concern cavity‐nesting *Hylaeus* and should not be generalized to the entire wild bee community, the positive association with organic farming is consistent with patterns reported using different sampling approaches and across bee communities with different nesting strategies.

Although our findings are consistent with those of Power and Stout (2011) in Ireland, who reported greater flower abundance and plant species richness at organic sites, our results should be interpreted with some caution. We similarly found greater floral resource availability at organic sites, supporting the view that contrasting farming practices can influence the local floral environment. However, because floral resources were surveyed only once, we refrained from attributing population‐level responses of *Hylaeus* directly to the observed differences in flower availability. Although our survey was conducted in July, during the main activity period of *H. communis* (Westrich 1989), floral resources are highly dynamic, and a single survey may not represent the resources available throughout the species' entire active period. In contrast, farming management remained consistent throughout this period and may therefore better represent the broader environmental conditions experienced by the bees.

For this reason, we considered farming system a more appropriate predictor for interpreting population‐level differences in *Hylaeus*, while the observed differences in floral resources provide a potential ecological mechanism through which farming management may indirectly affect bee populations. According to our findings, *H. communis* was indeed influenced by farming management, reinforcing previous studies (Holzschuh et al. 2008; Kennedy et al. 2013; Williams and Kremen 2007) on larger sized bees indicating that organic farming is more sustainable and helps for resilience of wild bees in agricultural landscapes (BMEL 2024).

The negative relationship between forest cover and the abundance of *Hylaeus* may reflect the ecological association of these bees with relatively open habitats. *Hylaeus* commonly forage on herbaceous flowering plants (Koster 1986; Martins et al. 2017; Westrich 1989), which may be more abundant in open agricultural habitats and field margins than in closed forest. Alternatively, greater forest cover may increase the availability of natural nesting substrates (Mullally et al. 2019; Winfree et al. 2007), potentially reducing the use of artificial trap nests. Thus, the observed decline in *Hylaeus* abundance with increasing forest cover may reflect differences in both foraging habitat and nesting opportunities.

### 4.2 *H. communis* Sex Ratio

Although approximately equal investment in male and female offspring may be expected under favorable and resource‐abundant conditions (Peterson et al. 2006; Westrich 2019), male‐biased sex ratios are commonly reported in wild bee populations (Bosch et al. 2021; Gruber et al. 2011). Differences in the energetic costs of producing the two sexes may contribute to such variation, as female offspring are generally more costly to produce than males (Peterson et al. 2006; Torchio and Tepedino 1980). Consequently, local resource conditions and other environmental factors experienced by nesting females may influence maternal investment and offspring sex allocation. Environmental stressors may also affect this balance. For example, pesticide exposure has been associated with male‐biased offspring production in *Osmia lignaria* (Stuligross and Williams 2021) and *O. cornuta* (Strobl et al. 2019), potentially reflecting maternal responses to environmental conditions.

The ecological consequences of investment in the two sexes may also differ. Males can potentially mate with several females and disperse from their natal sites (Paxton 2005), whereas females of many solitary bees exhibit greater philopatry and establish nests within or near their natal populations. Consequently, although both sexes contribute to reproduction, female offspring may contribute more directly to the persistence and growth of the local nesting population (Paxton 2005; Staab et al. 2018).

In our study, the greater relative production of males at organic sites may be associated with the substantially larger *Hylaeus* population under organic management. Under more favorable conditions, increased reproductive output could permit greater production of the less costly male offspring while maintaining sufficient female production for local population persistence. Alternatively, because female bees can control offspring sex through fertilization (Zayed and Packer 2005), the relatively greater representation of females at conventional sites could reflect differential maternal sex allocation under contrasting environmental conditions. Despite their higher production costs (Peterson et al. 2006; Torchio and Tepedino 1980), maintaining female production may be particularly important for local population persistence because females establish the next generation of nests. These interpretations should nevertheless be treated as alternative hypotheses.

The sex ratio became significantly more female‐biased with increasing forest cover. Several non‐exclusive mechanisms could explain this pattern. First, forests may provide relatively stable and less intensively managed habitats, with substantially lower direct exposure to agricultural inputs such as fertilizers and pesticides than cropped areas (Barroso et al. 2022). Second, greater forest cover may increase landscape heterogeneity and the availability of forest edges, where woody vegetation and adjacent open habitats can provide complementary nesting and foraging resources (Karnchananiyom et al. 2024). Forested landscapes may also contain flower‐rich microhabitats and a greater diversity of potential nesting substrates, including pre‐existing cavities in dead wood, stems, and trees that can be used by cavity‐nesting bees (Mullally et al. 2019; Winfree et al. 2007).

Interestingly, this pattern may also provide an alternative explanation for the negative relationship between forest cover and the abundance of *Hylaeus* recorded in our study. Increasing forest cover could simultaneously provide additional natural nesting opportunities, reducing the use of artificial nests, while supporting conditions favorable to female production or persistence. Because females are responsible for nest establishment and may exhibit stronger natal‐site fidelity than males, greater forest cover could potentially contribute to the maintenance of local nesting populations even when fewer individuals are recorded in artificial nests. However, our data cannot distinguish between changes in offspring sex allocation and differences in habitat use, dispersal, or survival between the sexes. Future studies comparing *H. communis* nesting and sex ratios directly across forested and agricultural habitats would help determine the mechanisms underlying this relationship.

### 4.3 Effect of Farming System on Body Size

*Hylaeus* is among the many wild bee genera exhibiting sexual size dimorphism, with females generally being larger than males (Bosch and Vicens 2002). Larger female body size may be associated with greater energetic demands related to foraging, nest construction, provisioning, and reproduction (Michener 2007). In contrast, males do not provision nests or contribute to brood care and generally have lower energetic requirements associated with reproduction (Paxton 2005; Peterson et al. 2006). The larger size of female *H. communis* observed in our study is therefore consistent with the different reproductive and energetic demands experienced by the two sexes.

Although farming management has previously been associated with body‐size differences in larger bee species like *Osmia* (Boff et al. 2024), we found no evidence that organic versus conventional farming affected body size in the relatively small *H. communis* . This lack of response may be related to the ecological characteristics of the species. Floral resource abundance and diversity can differ substantially between farming systems, with organic farming generally supporting greater floral resources than conventional farming (Holzschuh et al. 2008). Changes in land use can consequently alter the quantity and composition of resources available to developing bees and potentially affect adult body size, with more specialized species potentially being particularly vulnerable to such changes (Anderson et al. 2024; Biesmeijer et al. 2006).

However, *H. communis* is considered a highly generalist bee species, capable of exploiting a broad range of floral resources and environmental conditions (Koster 1986; Martins et al. 2017; Westrich 1989). Such dietary flexibility, combined with the relatively low absolute resource requirements of this small‐bodied species, may buffer its development against differences in floral‐resource availability between farming systems. Thus, even if organic and conventional sites differ in flower abundance and composition, both environments may provide sufficient resources for *H. communis* larvae to attain a similar adult body size. This could explain why farming management affected other population‐level traits in our study but was not reflected in the body size of *H. communis* .

Nevertheless, similar adult body sizes do not necessarily indicate similar nutritional conditions. Individuals developing in organic and conventional landscapes may use different plant species and consequently receive pollen provisions differing in nutritional and chemical composition.

### 4.4 Effect of Farming System on the Composition of Cuticular Lipids

The composition of cuticular lipids of *H. communis* differed significantly between farming systems, with differences detected separately in both females and males. Organic and conventional farms differ in several management practices, including vegetation management and the use of plant‐protection products. Organic farming restricts the products that can be applied (BVL 2025; Haffmans and Ehrmantraut 2011), whereas conventional farming permits a broader range of synthetic fertilizers and pesticides (Montgomery and Biklé 2021).

Because small‐bodied *H. communis* have relatively short foraging ranges (Gathmann and Tscharntke 2002), their chemical phenotype may be particularly influenced by environmental conditions surrounding their nests. Bees foraging in conventionally managed fields and adjacent habitats may encounter pesticides through contaminated nectar and pollen orally (Zioga et al. 2023), or direct contact with agrochemicals (Fine et al. 2017), potentially affecting metabolic pathways involved in lipid biosynthesis. Conversely, organic farms may provide greater floral resource availability (Power and Stout 2011), although pesticide drift from surrounding areas may still occur (Alexander et al. 2021; Aude et al. 2003). Thus, the observed differences should be interpreted as responses to contrasting farming environments rather than as direct evidence of pesticide effects. Nevertheless, our findings are consistent with experimental and landscape‐level studies showing that agricultural stressors and farming management can alter cuticular lipid profiles in wild bees (Boff et al. 2024; Boff and Ayasse 2024; Mayr et al. 2025).

Interestingly, the separation between farming systems was visually more pronounced in females than in males, consistent with previous observations in female *O. bicornis* (Boff et al. 2024). Because cuticular lipids can contribute to chemical communication and mate recognition, changes in female chemical profiles could potentially influence mating and reproductive success. Although this mechanism was not directly tested here, it provides a compelling hypothesis linking the altered chemical profiles with the lower *Hylaeus* population size observed at conventional sites and deserves experimental investigation in *H. communis* .

## 5 Conclusion

Our findings indicate that farming management is an important factor shaping populations of small wild bees in agricultural landscapes. Organic farming appears to provide conditions that better support *Hylaeus*, highlighting its potential contribution to the conservation of wild bees in intensively managed landscapes. Increasing the extent of organic farming within predominantly conventional agricultural areas may therefore enhance their value for wild bee conservation. In this context, Germany's goal of reaching 30% organic agricultural land by 2030 represents an important opportunity to promote more bee‐friendly agricultural landscapes and biodiversity conservation.

## Author Contributions

**Sara Olberz:** data curation (equal), formal analysis (equal), investigation (equal), methodology (equal), validation (equal), visualization (equal), writing – original draft (equal). **Steven Jansen:** investigation (supporting), resources (supporting), supervision (supporting), validation (supporting), visualization (supporting), writing – original draft (supporting). **Manfred Ayasse:** investigation (supporting), methodology (supporting), resources (supporting), supervision (supporting), validation (supporting), visualization (supporting), writing – original draft (supporting). **Samuel Boff:** conceptualization (lead), data curation (equal), formal analysis (lead), funding acquisition (lead), investigation (lead), methodology (lead), project administration (lead), resources (lead), supervision (lead), validation (lead), visualization (lead), writing – original draft (supporting).

## Funding

We are grateful for the funding of Aurelia Stiftung and Software AG—Stiftung (SAGST).

## Conflicts of Interest

The authors declare no conflicts of interest.

## Supporting information


**Figure S1:** Example for measuring the intertegular distance (ITD), meaning the white line between the wing bases, in μm of a *H. communis* in a wax mold on a glass slide.
**Figure S2:** Number of *Hylaeus* nests in relation to (a) forest cover and (b) farming system (conventional and organic). In (a), the line represents the model‐predicted relationship between forest cover and the number of *Hylaeus* nests, with the shaded area indicating the 95% confidence interval. In (b), boxes represent the interquartile range (IQR), horizontal lines indicate the median, and whiskers extend to the most extreme values within 1.5 × IQR.
**Figure S3:** Number of *Hylaeus* bees in relation to (a) the extent of forest and (b) farming system. The box plot ranges from the first to the third quartile, representing the IQR, and includes the median, the middle value of the data. The extending whiskers represent the range of variation. The effect of farming system on the number of *Hylaeus* bees was marginally non‐significant.
**Figure S4:** Gas chromatogram of the extracted cuticular lipids of a (a) male and (b) female *H. communis* per time [min]. The components were separated using a non‐polar column in the GC and labeled peaks were identified by alkane, respectively alkene raster, reference mixtures, and by using mass spectrometry (for the list of chemical compounds see Table S5, C16 marks additionally the internal standard).
**Figure S5:** NMDS of cuticular lipids of individual bouquets of male (♂) and female (♀) *H. communis* from organic and conventional farming sites (*R* ^2^ = 0.948). All coordinates are connected to the centroids of the ellipses, each representing one type of farming management.
**Table S1:** The extent of organic farming [%] and extent of forest [%] per farming system and numbered site, calculated within a 500 m radius around the bee hotels. The numbering belongs to the map of farming sites (Figure 1).
**Table S2:** Average flower species and total flower abundance per farming system and numbered site from one‐time monitoring. The flowers within a 100 m radius around the bee hotels were monitored in 8 randomized plots of 25 m^2^. The numbering belongs to the map of farming sites (Figure 1).
**Table S3:** The number of *Hylaeus* and *H. communis* nests per farming system and numbered site. The numbering belongs to the map of farming sites (Figure 1).
**Table S4:** Effects of farming system and forest extent on genus‐level *Hylaeus* population parameters. Results from zero‐inflated negative‐binomial generalized linear mixed models (GLMMs; nbinom2, log link). Site was included as a random intercept. Effect estimates are presented with 95% confidence intervals.
**Table S5:** Mean body size of female and male *H. communis* sampled at organic and conventional farming sites. Values are presented as mean ± standard deviation (SD), with the number of individuals (*n*) measured in each group. Individuals with missing body‐size measurements were excluded from the calculations.
**Table S6:** Relative amounts of characteristic compounds of cuticular lipids [average ± standard deviation %] of *H. communis* from organic and conventional farming. *: Alkenes and alkadienes with unknown double bond position could not be confirmed.

## Data Availability

The data is available for peer review at dryad. http://doi.org/10.5061/dryad.1rn8pk18w.
