# Supplementary material for: Agricultural Landscapes and Forest Fragments Shape Population Dynamics, Cuticular Lipids and the Conservation of the Masked Bees, Hylaeus communis
Source: Ecol Evol. 2026 Oct 5;16(10):e74436. doi: 10.1002/ece3.74436 (PMC13639121; doi:10.1002/ece3.74436)
Supplement: Supplementary file 1 [file ECE3-16-e74436-s001.docx]

# **Supplementary material**

Figure S1. Example for measuring the intertegular distance (ITD), meaning the white line between the wing bases, in µm of a *H. communis* in a wax mold on a glass slide.

**Figure S2.** Number of Hylaeus nests in relation to **(a)** forest cover and **(b)** farming system (conventional and organic). In **(a)**, the line represents the model-predicted relationship between forest cover and the number of Hylaeus nests, with the shaded area indicating the 95% confidence interval. In **(b)**, boxes represent the interquartile range (IQR), horizontal lines indicate the median, and whiskers extend to the most extreme values within 1.5 × IQR.

Figure S3. **Number of** Hylaeus **bees in relation to a)** the extent of forest and **b)** farming system. The box plot ranges from the first to the third quartile, representing the IQR, and includes the median, the middle value of the data. The extending **whiskers** represent the **range of variation**. The effect of farming system on the number of Hylaeus **bees** was **marginally non-significant**.

**b**

**a**

**Figure S4.** Gas chromatogram of the extracted cuticular lipids of a a) male and b) female *H. communis* per time [min]. The components were separated using a non-polar column in the GC and labelled peaks were identified by alkane, respectively alkene raster, reference mixtures, and by using mass spectrometry (for the list of chemical compounds see Table S5, C16 marks additionally the internal standard).

**Figure S5.** NMDS of cuticular lipids of individual bouquets of male (♂) and female (♀) *H. communis* from organic and conventional farming sites (R^2^ = 0.948). All coordinates are connected to the centroids of the ellipses, each representing one type of farming management.

Table S1. The extent of organic farming [%] and extent of forest [%] per farming system and numbered site, calculated within a 500 m radius around the bee hotels. The numbering belongs to the map of farming sites (Figure 1).

| **Number** | **Farming system** | **Extent of organic farming** | **Extent of forest** |
| --- | --- | --- | --- |
| 1 | Organic | 69.03 | 50.23 |
| 2 | Organic | 84.25 | 12.54 |
| 3 | Organic | 54.41 | 36.17 |
| 4 | Organic | 69.18 | 14.24 |
| 5 | Organic | 44.40 | 27.00 |
| 6 | Organic | 94.42 | 28.75 |
| 7 | Organic | 46.34 | 39.11 |
| 8 | Organic | 88.55 | 31.10 |
| 9 | Organic | 57.83 | 16.33 |
| 10 | Conventional | 6.76 | 30.87 |
| 11 | Conventional | 3.78 | 37.26 |
| 12 | Conventional | 29.81 | 8.06 |
| 13 | Conventional | 1.75 | 11.03 |
| 14 | Conventional | 0.00 | 17.68 |
| 15 | Conventional | 6.49 | 19.83 |
| 16 | Conventional | 4.30 | 1.16 |
| 17 | Conventional | 0.00 | 42.89 |

**Table S2.** Average flower species and total flower abundance per farming system and numbered site from one-time monitoring. The flowers within a 100 m radius around the bee hotels were monitored in 8 randomized plots of 25 m^2^. The numbering belongs to the map of farming sites (Figure 1).

| **Number** | **Farming system** | **Average flower species** | **Total flower abundance** |
| --- | --- | --- | --- |
| 1 | Organic | 5.63 | 216.77 |
| 2 | Organic | 0.00 | 0.00 |
| 3 | Organic | 5.13 | 10.56 |
| 4 | Organic | 4.88 | 198.72 |
| 5 | Organic | 0.00 | 0.00 |
| 6 | Organic | 10.00 | 11.53 |
| 7 | Organic | 9.00 | 16.10 |
| 8 | Organic | 5.25 | 55.93 |
| 9 | Organic | 8.88 | 24.05 |
| 10 | Conventional | 0.00 | 0.00 |
| 11 | Conventional | 3.63 | 4.72 |
| 12 | Conventional | 1.38 | 4.33 |
| 13 | Conventional | 0.00 | 0.00 |
| 14 | Conventional | 1.75 | 3.61 |
| 15 | Conventional | 1.88 | 3.02 |
| 16 | Conventional | 0.25 | 2.00 |
| 17 | Conventional | 2.63 | 1.98 |

**Table S3.** The number of *Hylaeus* and *H. communis* nests per farming system and numbered site. The numbering belongs to the map of farming sites (Figure 1).

| **Number** | **Farming system** | ***Hylaeus* nests** | ***H. communis* nests** |
| --- | --- | --- | --- |
| 1 | Organic | 51 | 15 |
| 2 | Organic | 13 | 0 |
| 3 | Organic | 23 | 5 |
| 4 | Organic | 8 | 4 |
| 5 | Organic | 24 | 13 |
| 6 | Organic | 3 | 1 |
| 7 | Organic | 4 | 2 |
| 8 | Organic | 22 | 13 |
| 9 | Organic | 0 | 0 |
| 10 | Conventional | 0 | 0 |
| 11 | Conventional | 2 | 1 |
| 12 | Conventional | 4 | 2 |
| 13 | Conventional | 17 | 6 |
| 14 | Conventional | 4 | 3 |
| 15 | Conventional | 10 | 4 |
| 16 | Conventional | 14 | 10 |
| 17 | Conventional | 6 | 5 |

**Table S4.** Effects of farming system and forest extent on genus-level *Hylaeus* population parameters. Results from zero-inflated negative-binomial generalized linear mixed models (GLMMs; nbinom2, log link). Site was included as a random intercept. Effect estimates are presented with 95% confidence intervals.

| **Response** | **Predictor** | **Effect estimate** | **95% CI** | **χ²** | **df** | **p** |
| --- | --- | --- | --- | --- | --- | --- |
| *Hylaeus* nests | Forest extent | β = −3.98 | [−9.12, 1.16] | 0.86 | 1 | 0.355 |
| *Hylaeus* nests | Farming system | C/O ratio = 0.39 | [0.14, 1.05] | 3.41 | 1 | 0.065 |
| *Hylaeus* nests | System × Forest | β = 4.72 | [−2.81, 12.25] | 1.51 | 1 | 0.219 |
| *Hylaeus* bees | Forest extent | β = −5.54 | [−11.33, 0.25] | 1.41 | 1 | 0.235 |
| *Hylaeus* bees | Farming system | C/O ratio =0.34 | [0.11, 1.06] | 3.48 | 1 | 0.062 |
| *Hylaeus* bees | System × Forest | β = 6.49 | [−2.14, 15.13] | 2.17 | 1 | 0.141 |

Notes. C/O ratio = model-estimated conventional/organic ratio based on estimated marginal means, back-transformed to the response scale. β coefficients are reported on the conditional-model log scale. Wald χ² statistics and associated p-values are from Type II tests.

**Table S5.** Mean body size of female and male H. communis sampled at organic and conventional farming sites. Values are presented as mean ± standard deviation (SD), with the number of individuals (n) measured in each group. Individuals with missing body-size measurements were excluded from the calculations.

| **Farming system** | **Sex** | **n** | **Mean size** | **SD** |
| --- | --- | --- | --- | --- |
| Organic | Female | 34 | 1234.75 | 119.55 |
| Organic | Male | 82 | 1071.94 | 119.55 |
| Conventional | Female | 34 | 1248.08 | 116.05 |
| Conventional | Male | 35 | 1082.96 | 77.09 |

Table S6. Relative amounts of characteristic compounds of cuticular lipids [average ± standard deviation %] of *H. communis* from organic and conventional farming. *: Alkenes and alkadienes with unknown double bond position could not be confirmed.

| **Number** | **Compound** | **Organic** | **Conventional** |
| --- | --- | --- | --- |
| 1 | Unknown 1 | 1.274 ± 1.171 | 0.316 ± 0.592 |
| 2 | Unknown 2 | 3.455 ± 4.857 | 1.035 ± 2.143 |
| 3 | Unknown 3 | 1.826 ± 3.492 | 2.964 ± 5.741 |
| 4 | Unknown 4 | 10.467 ± 14.269 | 21.406 ± 14.990 |
| 5 | Heptadecane | 0.056 ± 0.088 | 0.241 ± 0.994 |
| 6 | Unknown 5 | 1.270 ± 1.743 | 1.853 ± 1.753 |
| 7 | Unknown 6 | 0.942 ± 1.923 | 2.018 ± 3.528 |
| 8 | Octadecane | 0.149 ± 0.205 | 0.314 ± 0.212 |
| 9 | Nonadecane | 0.139 ± 0.196 | 0.285 ± 0.187 |
| 10 | Unknown 7 | 0.930 ± 1.422 | 1.933 ± 2.760 |
| 11 | Eicosane | 0.175 ± 0.166 | 0.308 ± 0.199 |
| 12 | Unknown 8 | 0.396 ± 0.548 | 0.839 ± 0.578 |
| 13 | Heneicosene 1* | 0.150 ± 0.219 | 0.339 ± 0.255 |
| 14 | Heneicosane | 0.369 ± 0.388 | 0.698 ± 0.352 |
| 15 | Unknown 9 | 0.893 ± 0.921 | 0.575 ± 0.736 |
| 16 | Oleic acid | 4.449 ± 7.692 | 5.631 ± 9.307 |
| 17 | Unknown 10 | 1.015 ± 1.117 | 1.958 ± 1.050 |
| 18 | Docosane | 0.839 ± 0.932 | 1.230 ± 0.686 |
| 19 | Unknown 11 | 0.687 ± 0.853 | 1.456 ± 0.898 |
| 20 | Tricosene 1* | 0.076 ± 0.116 | 0.179 ± 0.124 |
| 21 | Tricosene 2* | 0.062 ± 0.101 | 0.093 ± 0.063 |
| 22 | Tricosane | 2.328 ± 1.928 | 2.662 ± 1.434 |
| 23 | Tetracosane | 2.698 ± 3.013 | 3.354 ± 2.264 |
| 24 | Pentacosene 1* | 0.818 ± 1.689 | 0.585 ± 0.837 |
| 25 | Pentacosene 2* | 0.195 ± 0.298 | 0.360 ± 0.243 |
| 26 | Pentacosene 3* | 0.113 ± 0.166 | 0.254 ± 0.169 |
| 27 | Pentocosane | 12.567 ± 5.626 | 8.176 ± 4.134 |
| 28 | Hexacosane | 4.230 ± 5.137 | 4.490 ± 3.202 |
| 29 | Unknown 12 | 0.344 ± 0.516 | 0.390 ± 0.385 |
| 30 | Heptacosene 1* | 1.693 ± 3.344 | 0.901 ± 1.289 |
| 31 | Heptacosene 2* | 0.372 ± 0.793 | 0.450 ± 0.441 |
| 32 | Heptacosene 3* | 0.050 ± 0.062 | 0.071 ± 0.157 |
| 33 | Heptacosane | 7.043 ± 3.378 | 5.325 ± 2.591 |
| 34 | Octacosane | 2.242 ± 2.951 | 2.216 ± 2.000 |
| 35 | Unknown 13 | 1.200 ± 1.720 | 2.338 ± 1.817 |
| 36 | Unknown 14 | 1.417 ± 2.324 | 0.709 ± 0.664 |
| 37 | Nonacosene 1* | 1.661 ± 2.062 | 1.053 ± 1.398 |
| 38 | Nonacosene 2* | 0.383 ± 0.392 | 0.479 ± 0.364 |
| 39 | Nonacosene 3* | 0.071 ± 0.114 | 0.080 ± 0.115 |
| 40 | Nonacosane | 2.763 ± 1.599 | 1.984 ± 1.221 |
| 41 | Triacontane | 0.885 ± 1.128 | 0.938 ± 0.820 |
| 42 | Hentriacontadiene 1* | 0.375 ± 0.305 | 0.294 ± 0.276 |
| 43 | Hentriacontadiene 2* | 0.347 ± 0.847 | 0.419 ± 0.411 |
| 44 | Hentriacontadiene 3* | 1.595 ± 2.204 | 0.794 ± 0.958 |
| 45 | Hentriacontene 1* | 2.210 ± 2.584 | 1.108 ± 1.427 |
| 46 | Hentriacontene 2* | 0.544 ± 0.541 | 0.413 ± 0.453 |
| 47 | Hentriacontene 3* | 0.208 ± 0.254 | 0.085 ± 0.111 |
| 48 | Hentriacontane | 1.522 ± 0.989 | 0.934 ± 0.513 |
| 49 | Unknown 15 | 0.243 ± 0.351 | 0.519 ± 0.526 |
| 50 | Dotriacontane | 0.238 ± 0.314 | 0.254 ± 0.235 |
| 51 | Unknown 16 | 0.999 ± 1.895 | 0.566 ± 0.890 |
| 52 | Unknown 17 | 1.120 ± 1.215 | 0.815 ± 0.725 |
| 53 | Unknown 18 | 2.862 ± 3.244 | 1.430 ± 2.248 |
| 54 | Unknown 19 | 2.315 ± 2.815 | 1.016 ± 1.310 |
| 55 | Tritriacontane | 0.485 ± 0.376 | 0.423 ± 0.358 |
| 56 | Unknown 20 | 1.411 ± 2.211 | 0.705 ± 1.069 |
